# Testosterone signaling pathways for reducing neuropathic pain in a rat model of spinothalamic tract lesion

**DOI:** 10.22038/ijbms.2024.78491.16968

**Published:** 2024

**Authors:** Abbas Alimoradian, Fatemeh Abbaszadeh, Masoumeh Jorjani

**Affiliations:** 1 Department of Pharmacology, School of Medicine, Arak University of Medical Sciences, Arak, Iran; 2 Neurobiology Research Center, Shahid Beheshti University of Medical Sciences, Tehran, Iran; 3 Department of Pharmacology, School of Medicine, Shahid Beheshti University of Medical Sciences, Tehran, Iran

**Keywords:** Astrocyte, Molecular signaling, Neuropathic pain, Spinal cord injury, Testosterone

## Abstract

**Objective(s):**

Most individuals who suffer from spinal cord injury (SCI) experience neuropathic pain, which currently has no effective treatment. In this study, we examined how testosterone affects neuropathic pain resulting from SCI.

**Materials and Methods:**

We administered three different doses of testosterone (4, 8, 16 mg/kg, intraperitoneal) to male rats after an electrolytic lesion of the spinothalamic tract. We then conducted behavioral tests, including open field and von Frey tests, within 28 days post-SCI. On day 28 after SCI, we analyzed spinal tissue using western blot to measure the levels of ionized calcium binding adaptor molecule 1 (Iba1), glial fibrillary acidic protein (GFAP), phospho-extracellular signal-regulated kinase (p-ERK1/2), and p-P38 at the injury site.

**Results:**

The results showed that testosterone significantly improved both motor activity and mechanical allodynia compared to the SCI-only group. Testosterone also inhibited microglia and astrocyte activation. Furthermore, testosterone significantly decreased p-P38 and p-ERK levels.

**Conclusion:**

The findings indicate that testosterone may alleviate SCI-induced neuropathic pain by inhibiting the activation of astrocytes and microglia, as well as suppressing MAPK signaling pathways.

## Introduction

Every year, between 250,000 to 500,000 people around the world suffer from spinal cord injury (SCI). Various reasons, such as accidents, can cause these injuries, falls, or acts of violence. Men are more likely to suffer from SCI than women. SCI is often associated with mobility issues, central pain syndrome, and metabolic abnormalities, which can ultimately lead to a decreased life expectancy and induction of depression (1). Central pain syndrome manifests as a continuous burning sensation that can be evoked by mechanical touch or temperature stimuli (2). The spinothalamic tract (STT) is an essential sensory pathway in the nervous system responsible for transmitting pain, temperature, and crude touch sensations to the somatosensory region of the thalamus. Studies have shown that preserved tissue bridges and the recovery of STT function are associated with the emergence and intensity of neuropathic pain after SCI (3, 4). Despite significant progress in understanding the molecular and cellular changes caused by SCI, current treatments for neuropathic pain are limited due to the complex pathophysiological mechanisms involved. Therefore, neuropathic pain remains one of the most challenging medical conditions to treat.

The development of new drugs is crucial for individuals suffering from neuropathic pain. In men, SCI can cause endocrine dysfunction, leading to changes in testosterone production. Studies have indicated that men with SCI generally have lower testosterone levels than healthy men (5, 6**). **Testosterone administration has been shown to enhance hearing and performance (7, 8), increase bone density, strength, and recovery, improve muscle tone, boost libido and energy levels (9), reduce anxiety and depression, and improve cognitive function (10).

Testosterone can cross the blood-brain barrier by binding to androgen receptors in the brain. This binding can prevent the growth of astrocytes and microglia, reducing the neuroinflammatory response and stimulating myelin regeneration and healing (11). These effects can help to reduce neuronal apoptosis (12). Additionally, testosterone therapy has been found to have some neuroprotective and neurotherapeutic effects (13) that can improve neuropathy and chronic pain, as well as the quality of life (14-16). However, researchers still need to conduct more studies to understand the underlying mechanisms of testosterone’s analgesic effects. In this study, we aimed to evaluate the mechanism of action of testosterone in reducing neuropathic pain after SCI.

## Materials

***Animals***

For this study, a total of 42 adult male Wistar rats weighing 250–280 g were used. The rats were housed in a controlled environment with 12-hour light and dark cycles and provided free food and water access. All procedures were approved by the Neuroscience Research Center ethics committee at Shahid Beheshti University of Medical Sciences (IR.SBMU.PHNS.REC.1394.57).

***Spinal cord injury***

The experiment involved administering a ketamine/xylazine mixture to rats at a dose of 60/20 mg/kg through an intraperitoneal injection. A procedure similar to the one used in a previous study (17) was applied to induce SCI with a few adjustments. Specifically, the spinal cord at the T8-T9 level was accessed through a laminectomy, targeting the spinothalamic tract (STT).

During the surgical procedure, the protective membrane around the spinal cord called the dura, was cut. The electronic device used in this study was the UGO Basile model 53500 from Italy, which was connected to a tungsten electrode with a 5 µm tip and 1 MΩ resistance. The electrode was inserted into the right STT according to stereotaxic integration at a depth of 1.6-1.9 mm and a midline lateral position of 0.5–0.7 mm. A pulse current of 300 μA for 60 sec was passed through the electrodes to produce unilateral lesions. Following the surgery, each animal was given a single dose of antibiotics to prevent potential infections from surgery and sutures. Additionally, animals receive about 1 ml of sterile normal saline (S.C.) during surgery or post-surgery to maintain electrolyte balance in the case of intraoperative bleeding and dehydration.

***Experimental groups***

The rats in this study were divided into four main groups. The first group, known as the sham group (n=7), only underwent laminectomy without an electrolytic lesion. The second group, known as the injury group (n=7), was subjected to an electrical lesion. The third group, known as the injury+vehicle group (n=7), received an electrolytic lesion and 0.5 ml of sesame oil as a vehicle. Lastly, the treatment group was divided into three subgroups (n=7 in each group) and given different doses of testosterone (Sigma–Aldrich, St. Louis, MO, USA) (4, 8, and 16 mg/kg) via intraperitoneal injection 30 min after the injury. 

***Open field test***

To evaluate the rats’ movement activity, a black wooden box measuring 60x60x40 cm was used. The rats were allowed to explore the environment for 30 min after which they were placed in one corner of the box and given 5 min to move around freely. During each trial, the distance and velocity of each mouse were recorded using the Ethovision software package (version 7, Noldus Information Technology, the Netherlands).

***Mechanical allodynia***

In order to evaluate the mechanical allodynia, von Frey filaments were used. The filaments were applied in ascending order to the dorsal surface of the hind paw of both legs, between the second and third toes of the paw. The paw withdrawal threshold was defined as the force at which the animal withdrew the paw to three out of the five stimuli applied. To calculate the final response, the average score from both legs was considered (18).

***Western blot analysis***

On Day 28, a western blotting analysis was conducted on the T8-T9 spinal segments at the lesion site, following a previously published protocol (19, 20). Total protein extract was obtained by homogenizing spinal cord tissue with a lysis buffer containing NaCl (150 mM), sodium deoxycholate (0.25%), Triton X-100 (0.1%), Tris-HCL (50 mM), SDS (0.1%), EDTA (1 mM), and protease inhibitor cocktail (1%). The tissue homogenate was then centrifuged at 12,000 rpm for 45 min at 4 °C, and the protein concentration was determined using the Bradford assay. The protein samples were loaded on SDS-polyacrylamide gel electrophoresis (SDS-PAGE) and transferred to a polyvinylidene difluoride (PVDF) membrane. The membranes were incubated with non-fat dry milk 5% w/v, primary antibodies (anti-Iba1, anti-GFAP, anti-p-ERK, anti-p-P38 (1:1000), and anti-β actin (1:10000)) and secondary antibodies (rabbit or mouse, diluted at 1:3000). Finally, the membranes were reacted with the enhanced chemiluminescence kit (ECL solution) and exposed to x-ray films. The intensities of the bands were quantified using the Image J software. 

***Statistical analysis***

The statistical analysis was conducted using GraphPad Prism 8.0 Software. The data underwent one-way and two-way ANOVA tests, followed by *post hoc* tests, including Tukey’s or Bonferroni’s. A significance level of *P*<0.05 was considered statistically significant. The results are presented as mean ± SEM.

## Results

***Motor activity***

**Figure 1:**
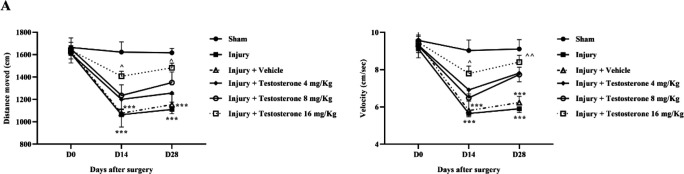
Effects of testosterone on motor function after spinal cord injury in rats

The analysis of the open field test data indicated that after the SCI, both the distance moved and velocity of movement in 5 min were significantly reduced on the 14th and 28th days (*P*<0.001). However, treatment with different doses of testosterone, especially at 16 mg/kg, significantly improved both parameters compared to the injury group (*P*<0.05) (Figure 1A & 1B). 

***Mechanical allodynia***

**Figure 2:**
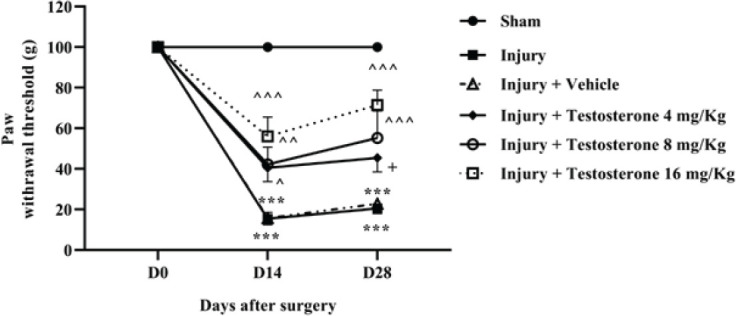
Effects of testosterone on mechanical pain after spinal cord injury in rats

At four weeks of follow-up, the sham group’s response to mechanical pain remained unchanged and was similar to that of day 0. However, spinothalamic pathway injury caused a significant and long-term reduction in the response threshold to mechanical stimulus, indicating mechanical allodynia on days 14 and 28 after the injury (*P*<0.001). Treatment with different doses of testosterone, particularly 16 mg/kg, significantly alleviated the symptoms caused by SCI and increased the response threshold to painful stimuli (*P*<0.001) (Figure 2). 

***GFAP and Iba1 expression***

**Figure 3:**
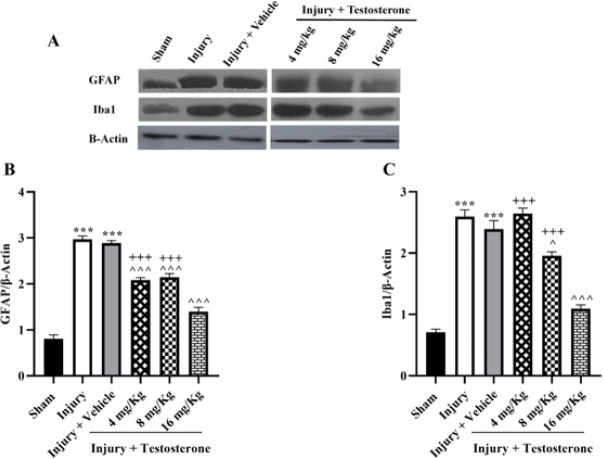
Effects of testosterone on expression levels of glial fibrillary acidic protein and Iba1 after spinal cord injury in rats

The levels of GFAP and Iba1 proteins in the spinal cord tissue after SCI were measured using western blot analysis (Figure 3A). The Injury group had significantly higher levels of GFAP and Iba1 proteins than the sham group (*P*<0.001). However, the administration of testosterone doses resulted in a significant reduction in the levels of these proteins, especially with a dose of 16 mg/kg (*P*<0.05, Figure 3B, C).

***MAPK signaling pathways***

**Figure 4:**
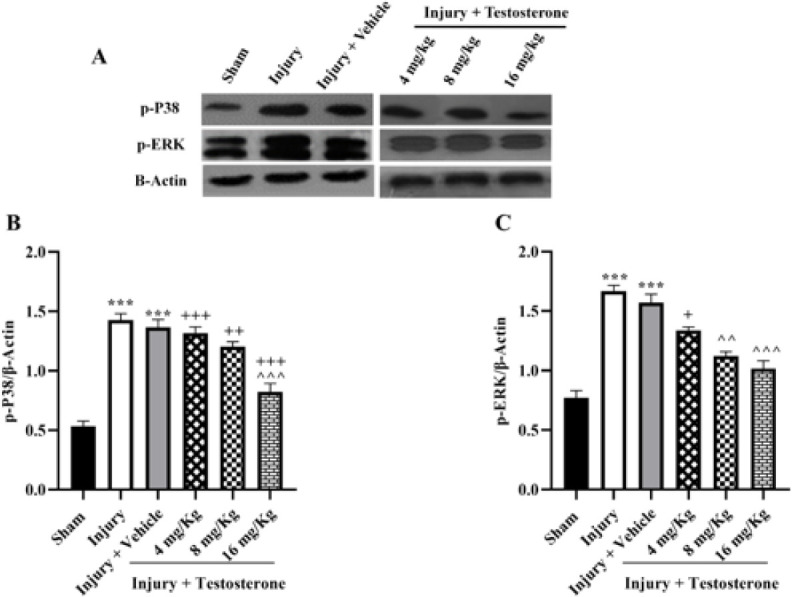
Effects of testosterone on expression levels of p-P38 and p-P38 after spinal cord injury in rats

Western blot analyses were conducted to determine the expression levels of p-P38 and p-ERK (Figure 4A). The results showed that after SCI, the expression levels of p-P38 (Figure 4B) and p-ERK (Figure 4C) were observed to be higher in spinal tissue than in the sham group. However, treatment with testosterone, especially at 16 mg/Kg, significantly reduced the increased expressions (*P*<0.01).

## Discussion

The present study aimed to investigate the effects of different doses of testosterone on changes in pain threshold, motor function, and inflammatory markers after a model of SCI. Following an electrolytic lesion on the STT, rats were administered varying doses of testosterone intraperitoneally. The findings revealed that testosterone, particularly at 16 mg/kg, was highly effective in reducing pain sensitivity after SCI in rats. This positive effect of testosterone was linked to decreased inflammatory markers of MAP-kinase subtypes, such as p-38 and p-ERK, and reduced astroglia activity, such as Iba1 and GFAP.

Neuropathic pain affects almost 53% of SCI patients (21). Unfortunately, there is no effective treatment available to reduce it. An appropriate animal model can help us understand the molecular mechanisms involved in neuropathic pain. The STT is the main pain tract, and destroying it unilaterally causes hyperalgesia and bilateral allodynia, which can last for weeks. These features are similar to those experienced by humans with central pain syndrome following SCI (22, 23). Therefore, this model can help to understand the main biological mechanisms of pain after SCI. 

Moreover, unlike the other SCI models, such as compression and contusion, which result in complete paralysis and require specialized care, such as urine drainage and daily administration of antibiotics to prevent post-surgery bacterial infection, the STT model does not require extensive. In this model, rats retain most of their normal reflexes, including bladder control, with no paralysis, and there is a lower rate of post-surgery infection than other SCI models. We found that administering a single dose of penicillin G effectively prevented bacterial infections, including those typically associated with surgical sites. There were no indications of urinary tract infections in the rats with SCIs either. 

The optimal time for drug administration for the treatment of SCI is also a subject of ongoing research and debate. Timing is crucial, and generally, the earlier the drug is administered, the better potential outcomes are expected. This is because an immediate cascade of events occurs after SCI, including inflammation, oxidative stress, and cell death. Early administration of a neuroprotective drug can prevent a cascade of cellular events in the acute phase and protect from progressive damage in the chronic phase. Several studies have suggested that the first few hours after injury are a critical time window for drug administration. There are reports that some neuroprotective drugs have shown better efficacy when given within the first few hours (24, 25). Therefore, in this study, testosterone was administered 30 min post-injury.

Our study found that administering a 16 mg/kg dose of testosterone significantly reduced mechanical allodynia and motor function deficits. Other studies in different animal models of SCI have shown that neurosteroids like testosterone can improve functional problems in SCI. They mainly act via autocrine and paracrine in the CNS (26), playing roles as neuroprotective, anti-apoptotic, anti-nociceptive, and anti-inflammatory agents (7, 27-29). A retrospective study shows that intramuscular injections of testosterone (200 mg, monthly) also improve motor function in SCI patients (30). Based on a report, lower testosterone levels (4.32 ng/ml) are associated with increased pain perception and activation of brain regions representing pain-related unpleasantness, but not pain intensity itself (31). Calabrese *et al*. also indicated that testosterone metabolites have analgesic properties for diabetic neuropathic pain, suggesting that they could be potential therapeutic agents for managing neuropathic pain in the clinic (15). 

Activation of microglia and astrocytes is known to be involved in the maintenance of neuropathic pain (32, 33). MAPK pathways such as p38 and ERK play a crucial role in the initiation and persistence of both central and peripheral neuropathic pain and are activated by glial cells (32, 34). Spinal cord glial cells are significantly activated by SCI, leading to the activation of MAPKs (35). A study reported that activated microglia enhance p-P38 and p-ERK (36). Additionally, it has been shown that intrathecal injection of SB203580 (as p38 inhibitor) or PD98059 (as ERK inhibitor) after SCI can effectively reduce mechanical and thermal pain threshold, indicating that the activation of p38 and ERK in microglia and astrocytes is implicated in the development of neuropathic pain following SCI (36).

Our results indicate that administering a 16 mg/kg dose of testosterone significantly reduces the expression levels of GFAP and Iba1 and the expression levels of p-ERK and p-P38 after SCI. It has been stated that testosterone exerts its effects through two main mechanisms. It can directly activate androgen receptors in different body tissues and can also be converted into other steroid hormones, such as androgen or estrogen, which play an essential role in the body. Both androgens and estrogens have been found to have neuroprotective properties in the nervous system (37). A study reported that estrogen inhibits neuropathic pain caused by SCI by inhibiting the activation of microglia and the activation of p38 and ERK in activated microglia in the spinal cord (38). Our previous studies have also demonstrated that estrogen inhibits neuropathic pain following SCI by suppressing glial cell activation (39) and decreasing glutamate levels in the ventral posterolateral nucleus (VPL) nuclei of rats (40). Testosterone administration has been shown to decrease GFAP expression and increase the recovery rate of facial muscle paralysis in hamsters following facial nerve injury (41). On the other hand, a decrease in circulating testosterone is associated with an age-related increase in GFAP (42).

Testosterone plays a crucial role in promoting neuroprotective signaling through several pathways. These pathways include regulation of transcription by nuclear medicine, non-genomic signaling via the MAP or phosphatidylinositol 3-kinase (PI3K) pathways, and non-receptor pathways through anti-oxidants scavenging free radicals (43, 44). Testosterone interacts directly with the MAP kinase pathway, which activates signaling molecules like Ras. This interaction activates various transcription factors that promote neuronal survival (45) and neuroprotection after neurotoxic events (46, 47). Testosterone reduces intracellular calcium and activates ERK in midbrain astroglia (48-50) while affecting the PI3-K/Akt signal transduction pathway (43, 51, 52). It plays a crucial role in promoting neuron survival and differentiation while providing protection against excitotoxicity and apoptosis. This is accomplished by triggering neurochemical changes in GABAergic neurons and opioid receptors in the spinal cord and periaqueductal gray (PAG) region. Ultimately, these changes reduce inflammatory mediators such as PGE2 and metalloproteinase-9 (51) as well as pain sensitivity (39). The MAPK/ERK pathway, activated by testosterone and its metabolites, is a major pathway for signaling related to cell growth, differentiation, and neuronal plasticity (53, 54). Carrier and Kabbaj reported that the MAPK signaling pathway mediates the effects of testosterone (55).

(A) Total distance moved and (B) velocity of movements. Data are shown as mean ± SEM (n=7). **P<0.01, ***P<0.001 versus sham; and ^*P*<0.05, ^^*P*<0.01 versus Injury+Vehicle group. Statistical comparisons were made using a two-way analysis of variance with the Bonferroni *post-hoc* test.

Data are shown as mean ± SEM (n=7). ***P<0.001 versus sham; ^^*P*<0.01, ^^^*P*<0.001 versus Injury+Vehicle group and +*P*<0.05 versus Injury+ Testosterone 16 mg/kg group. Statistical comparisons were made using a two-way analysis of variance with the Bonferroni* post-hoc *test.

(A) Western blot analysis and optical density ratio of (B) GFAP, (C) Iba1 to β-actin 28 days after SCI. Data are shown as mean ± SEM (n=3). **P<0.01, ***P<0.001 versus sham ^*P*<0.05, ^^^*P*<0.001 versus Injury+Vehicle group and +++*P*<0.001 versus Injury+ Testosterone 16 mg/kg group. Statistical comparisons were made using a one-way analysis of variance with the Tukey *post-hoc *test. GFAP: Glial fibrillary acidic protein, Iba1: Ionized calcium-binding adaptor molecule 1; SCI: Spinal cord injury.

(A) Western blot analysis and optical density ratio of (B) p-P38 and (C) p-ERK to β-actin 28 days after SCI. Data are shown as mean ± SEM (n=3). **P<0.01, ***P<0.001 versus sham; ^^*P*<0.01, ^^^*P*<0.001 versus Injury+Vehicle group and +*P*<0.05, ++*P*<0.01, +++*P*<0.001 versus Injury+ Testosterone 16 mg/kg group. Statistical comparisons were made using a one-way analysis of variance with the Tukey post-hoc test. p-ERK: Phosphorylated extracellular signal-regulated kinase; SCI: Spinal cord injury.

## Conclusion

Based on this research, it seems that administering a 16 mg/kg dose of testosterone through intraperitoneal injection can help alleviate neuropathic pain resulting from a unilateral STT lesion. The relief happens due to the inhibition of microglia and astrocyte activation, along with the suppression of MAPK signaling pathways.

## Data Availability

Data will be made available upon request.

## References

[1] Hearn JH, Cross A (2020). Mindfulness for pain, depression, anxiety, and quality of life in people with spinal cord injury: A systematic review. BMC Neurol.

[2] Watson JC, Sandroni P (2016). Central neuropathic pain syndromes. Mayo Clin Proc.

[3] Hari AR, Wydenkeller S, Dokladal P, Halder P (2009). Enhanced recovery of human spinothalamic function is associated with central neuropathic pain after SCI. Exp Neurol.

[4] Pfyffer D, Vallotton K, Curt A, Freund P (2020). Tissue bridges predict neuropathic pain emergence after spinal cord injury. J Neurol Neurosurg Psychiatry.

[5] Clark MJ, Schopp LH, Mazurek MO, Zaniletti I, Lammy AB, Martin TA (2008). Testosterone levels among men with spinal cord injury: relationship between time since injury and laboratory values. Am J Phys Med Rehabil.

[6] Hvarness H, Jakobsen H, Biering-Sørensen F (2007). Men with spinal cord injury have a smaller prostate than men without. Scand J Urol Nephrol.

[7] Wang H, Zhou W-x, Huang J-f, Zheng X-q, Tian H-j, Wang B (2020). Endocrine therapy for the functional recovery of spinal cord injury. Front Neurol.

[8] Christensen MD, Hulsebosch CE (1997). Chronic central pain after spinal cord injury. J Neurotrauma.

[9] Durga A, Sepahpanah F, Regozzi M, Hastings J, Crane DA (2011). Prevalence of testosterone deficiency after spinal cord injury. PM&R.

[10] Cunningham G, Matsumoto A, Swerdloff R, Chevy Chase (2003). Patient’s guide to low testosterone.

[11] Hussain R, Ghoumari AM, Bielecki B, Steibel J, Boehm N, Liere P (2013). The neural androgen receptor: a therapeutic target for myelin repair in chronic demyelination. Brain.

[12] Gürer B, Kertmen H, Kasim E, Yilmaz ER, Kanat BH, Sargon MF (2015). Neuroprotective effects of testosterone on ischemia/reperfusion injury of the rabbit spinal cord. Injury.

[13] Fargo KN, Foecking EM, Jones KJ, Sengelaub DR (2009). Neuroprotective actions of androgens on motoneurons. Front Neuroendocrinol.

[14] Kato Y, Shigehara K, Kawaguchi S, Izumi K, Kadono Y, Mizokami A (2020). Efficacy of testosterone replacement therapy on pain in hypogonadal men with chronic pain syndrome: A subanalysis of a prospective randomised controlled study in Japan (EARTH study). Andrologia.

[15] Calabrese D, Giatti S, Romano S, Porretta-Serapiglia C, Bianchi R, Milanese M (2014). Diabetic neuropathic pain: a role for testosterone metabolites. J Endocrinol.

[16] Tennant F, Lichota L (2010). Testosterone replacement in chronic pain patients. Pract Pain Manag.

[17] Ren K (1999). An improved method for assessing mechanical allodynia in the rat. Physiol Behav.

[18] Naseri K, Saghaei E, Abbaszadeh F, Afhami M, Haeri A, Rahimi F (2013). Role of microglia and astrocyte in central pain syndrome following electrolytic lesion at the spinothalamic tract in rats. J Mol Neurosci.

[19] Masoudi A, Dargahi L, Abbaszadeh F, Pourgholami MH, Asgari A, Manoochehri M (2017). Neuroprotective effects of astaxanthin in a rat model of spinal cord injury. Behav Brain Res.

[20] Burke D, Fullen BM, Stokes D, Lennon O (2017). Neuropathic pain prevalence following spinal cord injury: A systematic review and meta-analysis. Eur J Pain.

[21] Wang G, Thompson SM (2008). Maladaptive homeostatic plasticity in a rodent model of central pain syndrome: thalamic hyperexcitability after spinothalamic tract lesions. J Neurosci.

[22] Naseri K, Saghaei E, Abbaszadeh F, Afhami M, Haeri A, Rahimi F (2013). Role of microglia and astrocyte in central pain syndrome following electrolytic lesion at the spinothalamic tract in rats. J Mol Neurosci.

[23] Yoon DH, Kim YS, Young W (1999). Therapeutic time window for methylprednisolone in spinal cord injured rat. Yonsei Med J.

[24] Cadotte DW, Fehlings MG (2011). Spinal cord injury: A systematic review of current treatment options. Clin Orthop Relat Res.

[25] Schaeffer V, Meyer L, Patte-Mensah C, Mensah-Nyagan AG (2010). Progress in dorsal root ganglion neurosteroidogenic activity: Basic evidence and pathophysiological correlation. Prog Neurobiol.

[26] Coronel MF, Labombarda F, González SL (2016). Neuroactive steroids, nociception and neuropathic pain: A flashback to go forward. Steroids.

[27] Brotfain E, E Gruenbaum S, Boyko M, Kutz R, Zlotnik A, Klein M (2016). Neuroprotection by estrogen and progesterone in traumatic brain injury and spinal cord injury. Curr Neuropharmacol.

[28] Elkabes S, Nicot AB (2014). Sex steroids and neuroprotection in spinal cord injury: A review of preclinical investigations. Exp Neurol.

[29] Clark MJ, Petroski GF, Mazurek MO, Hagglund KJ, Sherman AK, Lammy AB (2008). Testosterone replacement therapy and motor function in men with spinal cord injury: A retrospective analysis. Am J Phys Med Rehabil.

[30] Choi JC, Park YH, Park SK, Lee JS, Kim J, Choi JI (2017). Testosterone effects on pain and brain activation patterns. Acta Anaesthesiol Scand.

[31] Hains BC, Waxman SG (2006). Activated microglia contribute to the maintenance of chronic pain after spinal cord injury. J Neurosci.

[32] Hulsebosch CE (2008). Gliopathy ensures persistent inflammation and chronic pain after spinal cord injury. Exp Neurol.

[33] Ji R-R, Woolf CJ (2001). Neuronal plasticity and signal transduction in nociceptive neurons: implications for the initiation and maintenance of pathological pain. Neurobiol Dis.

[34] Ji RR, Gereau RWt, Malcangio M, Strichartz GR (2009). MAP kinase and pain. Brain Res Rev.

[35] Choi DC, Lee JY, Lim EJ, Baik HH, Oh TH, Yune TY (2012). Inhibition of ROS-induced p38MAPK and ERK activation in microglia by acupuncture relieves neuropathic pain after spinal cord injury in rats. Exp Neurol.

[36] Byers JS, Huguenard AL, Kuruppu D, Liu NK, Xu XM, Sengelaub DR (2012). Neuroprotective effects of testosterone on motoneuron and muscle morphology following spinal cord injury. J Comp Neurol.

[37] Lee JY, Choi HY, Ju B-G, Yune TY (2018). Estrogen alleviates neuropathic pain induced after spinal cord injury by inhibiting microglia and astrocyte activation. Biochim Biophys Acta.

[38] Saghaei E, Abbaszadeh F, Naseri K, Ghorbanpoor S, Afhami M, Haeri A (2013). Estradiol attenuates spinal cord injury-induced pain by suppressing microglial activation in thalamic VPL nuclei of rats. Neurosci Res.

[39] Naderi A, Asgari AR, Zahed R, Ghanbari A, Samandari R, Jorjani M (2014). Estradiol attenuates spinal cord injury-related central pain by decreasing glutamate levels in thalamic VPL nucleus in male rats. Metab Brain Dis.

[40] Coers S, Tanzer L, Jones KJ (2002). Testosterone treatment attenuates the effects of facial nerve transection on glial fibrillary acidic protein (GFAP) levels in the hamster facial motor nucleus. Metab Brain Dis.

[41] Day JR, Frank AT, O’Callaghan JP, Jones BC, Anderson JE (1998). The effect of age and testosterone on the expression of glial fibrillary acidic protein in the rat cerebellum. Exp Neurol.

[42] Ivanova T, Méndez P, Garcia-Segura LM, Beyer C (2002). Rapid stimulation of the PI3-kinase/Akt signalling pathway in developing midbrain neurones by oestrogen. J Neuroendocrinol.

[43] Honda K, Sawada H, Kihara T, Urushitani M, Nakamizo T, Akaike A (2000). Phosphatidylinositol 3-kinase mediates neuroprotection by estrogen in cultured cortical neurons. J Neurosci Res.

[44] Cooke BM, Breedlove SM, Jordan CL (2003). Both estrogen receptors and androgen receptors contribute to testosterone-induced changes in the morphology of the medial amygdala and sexual arousal in male rats. Horm Behav.

[45] Nguyen TVV, Yao M, Pike CJ (2005). Androgens activate mitogen-activated protein kinase signaling: Role in neuroprotection. J Neurochem.

[46] Hammond J, Le Q, Goodyer C, Gelfand M, Trifiro M, LeBlanc A (2001). Testosteronemediated neuroprotection through the androgen receptor in human primary neurons. J Neurochem.

[47] Ray SK, Samantaray S, Smith JA, Matzelle DD, Das A, Banik NL (2011). Inhibition of cysteine proteases in acute and chronic spinal cord injury. Neurotherapeutics.

[48] Dominguez R, Jalali C, de Lacalle S (2004). Morphological effects of estrogen on cholinergic neurons in vitro involves activation of extracellular signal-regulated kinases. J Neurosci.

[49] Aronson D, Violan MA, Dufresne SD, Zangen D, Fielding RA, Goodyear LJ (1997). Exercise stimulates the mitogen-activated protein kinase pathway in human skeletal muscle. J Clin Invest.

[50] Garcia-Ovejero D, Azcoitia I, Doncarlos LL, Melcangi RC, Garcia-Segura LM (2005). Glia-neuron crosstalk in the neuroprotective mechanisms of sex steroid hormones. Brain Res Rev.

[51] Wilson ME, Liu Y, Wise PM (2002). Estradiol enhances Akt activation in cortical explant cultures following neuronal injury. Mol Brain Res.

[52] Chen Z, Gibson TB, Robinson F, Silvestro L, Pearson G, Xu B-e (2001). MAP kinases. Chem Rev.

[53] Sweatt JD (2001). The neuronal MAP kinase cascade: A biochemical signal integration system subserving synaptic plasticity and memory. J Neurochem.

[54] Carrier N, Kabbaj M (2012). Extracellular signal-regulated kinase 2 signaling in the hippocampal dentate gyrus mediates the antidepressant effects of testosterone. Biol Psychiatry.

